# Synthetic lethal interactions of DEAD/H-box helicases as targets for cancer therapy

**DOI:** 10.3389/fonc.2022.1087989

**Published:** 2023-01-26

**Authors:** Ananna Bhadra Arna, Hardikkumar Patel, Ravi Shankar Singh, Frederick S. Vizeacoumar, Anthony Kusalik, Andrew Freywald, Franco J. Vizeacoumar, Yuliang Wu

**Affiliations:** ^1^ Department of Biochemistry, Microbiology and Immunology, University of Saskatchewan, Saskatoon, SK, Canada; ^2^ Department of Pathology and Laboratory Medicine, University of Saskatchewan, Saskatoon, SK, Canada; ^3^ Department of Computer Science, University of Saskatchewan, Saskatoon, SK, Canada; ^4^ Division of Oncology, College of Medicine, University of Saskatchewan and Saskatchewan Cancer Agency, Saskatoon, SK, Canada

**Keywords:** DEAD/H-box helicase, synthetic lethality, synthetic dosage lethality, therapeutic target, drug development, cancer

## Abstract

DEAD/H-box helicases are implicated in virtually every aspect of RNA metabolism, including transcription, pre-mRNA splicing, ribosomes biogenesis, nuclear export, translation initiation, RNA degradation, and mRNA editing. Most of these helicases are upregulated in various cancers and mutations in some of them are associated with several malignancies. Lately, synthetic lethality (SL) and synthetic dosage lethality (SDL) approaches, where genetic interactions of cancer-related genes are exploited as therapeutic targets, are emerging as a leading area of cancer research. Several DEAD/H-box helicases, including DDX3, DDX9 (Dbp9), DDX10 (Dbp4), DDX11 (ChlR1), and DDX41 (Sacy-1), have been subjected to SL analyses in humans and different model organisms. It remains to be explored whether SDL can be utilized to identity druggable targets in DEAD/H-box helicase overexpressing cancers. In this review, we analyze gene expression data of a subset of DEAD/H-box helicases in multiple cancer types and discuss how their SL/SDL interactions can be used for therapeutic purposes. We also summarize the latest developments in clinical applications, apart from discussing some of the challenges in drug discovery in the context of targeting DEAD/H-box helicases.

## Introduction

Helicases constitute a ubiquitous group of molecular motors that couple the energy from nucleoside triphosphate hydrolysis with the unwinding and/or remodeling of DNA or RNA molecules, and occasionally, with the disruption of protein-nucleic acid interactions. The human genome encodes 95 known helicases; out of them, 64 are RNA helicases and 31 are DNA helicases (1). These enzymes are involved in virtually all aspects of nucleic acid metabolism, including replication, repair, recombination, transcription, splicing, chromosome segregation and telomere maintenance (2–5). To support augmented proliferation and match the requirements of accelerated nucleic acid metabolism, helicases are frequently overexpressed in cancer cells (6). Meantime, naturally occurring loss of function (LOF) mutations in helicases are associated with many diseases, including cancers (7). Therefore, helicases become attractive targets for chemotherapeutic developments. Unfortunately, direct targeting of these molecules may represent a serious challenge, as normal cells are also highly dependent on their cellular functions. Therefore, alternate strategies are required.

Given that many helicases are overexpressed or lost in cancers, identifying their synthetic lethal (SL) and synthetic dosage lethal (SDL) interactions may represent an effective strategy to exploit them for cancer therapeutics. Two genes are said to exhibit SL interaction, if LOF of both these genes affect cellular viability, while neither of them has any effect on their own (8). This concept facilitates the development of targeted therapies that will selectively kill cancer cells, while sparing normal cells. Over the last decade, the most successful clinical application in the field of SL is the development of poly (ADP-ribose) polymerase-1 (PARP-1) inhibitors in BRCA1/2-mutant breast and ovarian cancers (9). In this review, we will highlight the potential of DEAD/H-box helicase in the invention of cancer therapeutics using the SL approach. We will also discuss potential opportunities to implement the SDL approach, where LOF of one gene affects cell viability only when a partner gene is overactivated, to exploit overexpressed DEAD/H-box helicases for cancer therapeutics.

## DEAD/H-box helicases

Based on substrate specificity and polarity, helicases are classified as RNA or DNA helicases and as 5ʹ–3ʹ or 3ʹ–5ʹ helicases (10). Based on their conserved motifs, helicases are grouped into six superfamilies (SF1 - SF6) (11). Among them, SF2 is the largest superfamily and is characterized by its 12 “signature” motifs (Q, I, Ia, Ib, Ic, II, III, IV, IVa, V, Va, and VI). SF2 is further classified into several subfamilies, including DEAD/H-box RNA helicases, RecQ-like family, and Snf2-like enzymes based on their sequences, structures, and mechanisms of action (12, 13).

**Figure 1:**
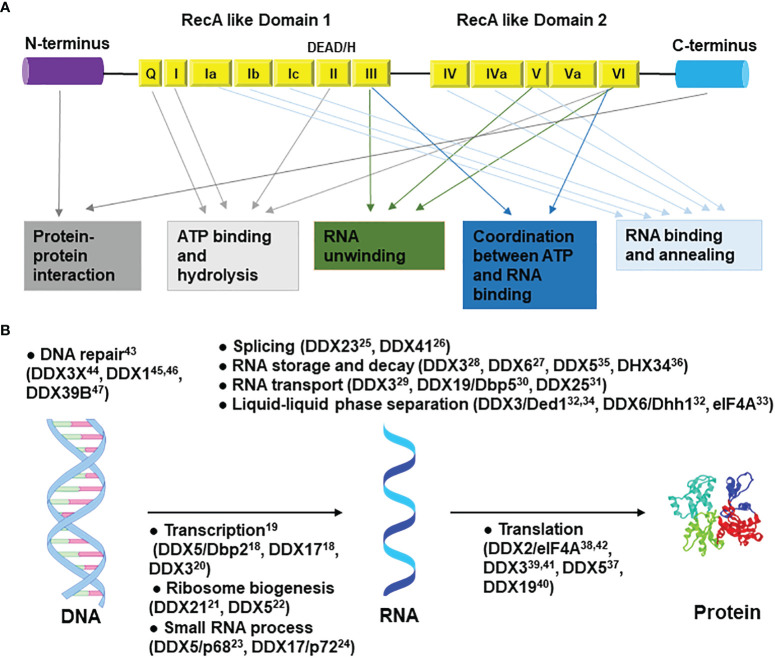
General structure and functions of DEAD/H-box helicases. **(A)** The N-terminal and C-terminal regions are involved in protein-protein interactions. Motifs Q, I, II, and VI are involved in ATP binding and hydrolysis; motifs III, V, and VI in RNA unwinding; motifs Ia, Ib, Ic, IV, IVa, and V in RNA binding and annealing; motifs III and Va in coordination between ATP and RNA binding. **(B)** Roles of DEAD/H-box RNA helicases in the central dogma pathway. Only the most studied top two to four helicases are shown in each catalogue. Some roles outside of the central dogma are not shown. Related references are shown as superscripts.

The DEAD/H-box protein family is named after the sequence (Asp-Glu-Ala-Asp/His) in motif II (14). They are all composed of two RecA-like domains, while some are additionally flanked by N- and/or C-terminal accessory domain(s) (**Figure 1A**). Cooperatively, these domains are involved in RNA binding, ATP binding and hydrolysis, unwinding, strand annealing, and protein-protein interactions (15). There are at least 36 DEAD-box helicases and 14 DEAH-box helicases in humans (16). Despite sharing the conserved helicase core, a substantial difference is observed between DEAD-box and DEAH-box helicases from the biochemical perspective. To disrupt nucleic acid structures, DEAD-box helicases use simple cycles of RNA duplex binding, unwinding, and release, while DEAH-box helicases function only as translocases in the 3’→5’ direction (17). In terms of their nucleic acid-related functions, both DEAD-box and DEAH-box proteins are implicated in virtually every aspect of RNA metabolic processes, including transcription (18–20), ribosomes biogenesis (21, 22), small RNA process (23, 24), pre-mRNA splicing (25, 26), RNA storage and decay (27, 28), nuclear export (29–31), liquid–liquid phase separation (32–34), RNA degradation (35, 36), translation (37–42), and so on (**Figure 1B**). Some of them are also involved in DNA metabolism, such as DNA repair (43–47). On a biological level, they are involved in innate immunity responses (48), signal transduction (49), cell differentiation and organ development (50, 51), programmed cell death (52), and mitochondrial regulations (53). Dysregulation of the expression or function of these proteins is likely to be one of the reasons behind the development of cancer and various diseases. For instance, mutations in DDX11 (ChlR1) are associated with Warsaw Breakage syndrome (WBS) and Roberts syndrome (54). Germline mutations in DDX3 account for 1%-3% of unexplained intellectual disability cases (55–59). Mutations in DDX6 cause intellectual disability and dysmorphic features (60). Mutations in DDX41 are associated with myelodysplastic syndromes (MDS), acute myeloid leukemia (AML), and myeloid neoplasms (MNs) (61, 62).

## Abnormal expression of DEAD/H-box helicases in cancer

To achieve the hallmark of excessive cell proliferation, DEAD/H-box helicases are often overexpressed in cancer cells (**Table 1**). For example, DDX3 is highly expressed in breast cancer (124), Ewing sarcoma (68), glioblastoma (125) and gallbladder carcinoma (126). DDX27 is upregulated in gastric tumors (94), colorectal cancer (127), and breast cancer (128). Cancer-specific antigens, DDX43 (helicase antigen gene, HAGE) and DDX53 (cancer-associated gene, CAGE), are overexpressed in almost all cancers (129), while DDX20 (dp103) is highly expressed in breast cancer (130). Nevertheless, controversial findings are reported for some DEAD/H-box helicases. For example, although DDX21 is highly expressed in breast cancer (131), colorectal cancer (89), gastric cancer (132) and neuroblastoma (90), low DDX21 levels are associated with poor clinical outcome of breast cancer patients (91). Moreover, up-regulation of DEAD/H-box helicases correlates with advanced stage and poor prognosis in cancer patients (7). For example, high DDX1 levels are associated with higher pathological grades and poorer prognosis in hepatocellular carcinoma (63) and breast cancer (133). The expression of DDX23 is enhanced in glioma tissues, and this is associated with poor survival of glioma patients (92). In computational analyses of 15 DEAH-box RNA helicases, elevated expression of 12 of them are associated with poor prognosis and worse clinical features in hepatocellular carcinoma (111). Because of their overarching role in RNA metabolism, DEAD/H-box helicases are likely to affect multiple aspects of cell behaviors, including cell proliferation, which may lead to cancer development.

**Table 1:** Abnormal expression of DEAD/H-box helicases in cancers.

Helicase^a^	Linked cancers^b^	Upregulated (Ref.)^c^	Notes
DDX1	Breast cancer, neuroblastoma, hepatocellular carcinoma	(63, 64)	
DDX2 (eIF4A)	Various cancers	(65, 66)	Downregulation is reported in non-small-cell lung cancer (67)
DDX3X	Various cancers	(68, 69)	Downregulation is reported in colorectal cancer (70) and hepatocellular carcinoma (71). DDX3 has two paralogs: DDX3X and DDX3Y.
DDX4 (Vasa)	Ovarian cancer	(72, 73)	
DDX5 (p68)	Various cancers	(74, 75)	
DDX6 (RCK/p54)	Gastric cancer, colorectal cancer	(76, 77)	
DDX10	Various cancers	(78, 79)	
DDX11 (ChlR1)	Renal cell carcinoma, lung adenocarcinoma	(80, 81)	Mutations are associated with WBS (54)
DDX17 (p72)	Various cancers	(82, 83)	
DDX18	Gastric cancer, esophageal squamous cell carcinoma	(84, 85)	Mutations found in AML/MDS patients (86)
DDX20	Various cancers	(87, 88)	
DDX21	Various cancers	(89, 90)	Downregulation is reported in breast cancer (91)
DDX23	Ovarian cancer, glioma	(92, 93)	
DDX27	Various cancers	(94, 95)	
DDX31	Pancreatic ductal adenocarcinoma, bladder cancer, renal cell carcinoma	(96, 97)	
DDX39B (BAT1)	Various cancers	(98, 99)	DDX39 has two paralogs: DDX39A and DDX39B
DDX43 (HAGE)	Various cancers	(100, 101)	Cancer-testis (CT) antigen
DDX49	Hepatocellular carcinoma, lung cancer	(102, 103)	
DDX53 (CAGE)	Various cancers	(104, 105)	Cancer-testis (CT) antigen
DDX56	Gastric cancer, colorectal cancer, Osteosarcoma	(106, 107)	
DDX58 (RIG-I)	Ovarian cancer, hepatocellular carcinoma	(108, 109)	RIG-I is well known as an RNA sensor against RNA viruses; Germline RIG-I mutations found in colon cancer (110)
DHX9 (RHA)	Prostate cancer, lung cancer, colorectal cancer, hepatocellular carcinoma	(111, 112)	
DHX15	AML, prostate cancer, Burkitt lymphoma	(113, 114)	
DHX32	Hepatocellular carcinoma, breast cancer, colorectal cancer	(115, 116)	Downregulation is reported in AML (117)
DHX33	Colon cancer, Glioblastoma, hepatocellular carcinoma	(118, 119)	
DHX36 (G4R1, RHAU)	Colon cancer, breast cancer, lung cancer	(120, 121)	
DHX37	Hepatocellular carcinoma	(122, 123)	

^a^Helicases are listed in an ascending order, and only helicases that have been reported by at least two independent groups (in PubMed) are included, ^b^Various cancers stands for more than three different cancers, and ^c^Two latest or the most representative references are cited.

**Figure 2:**
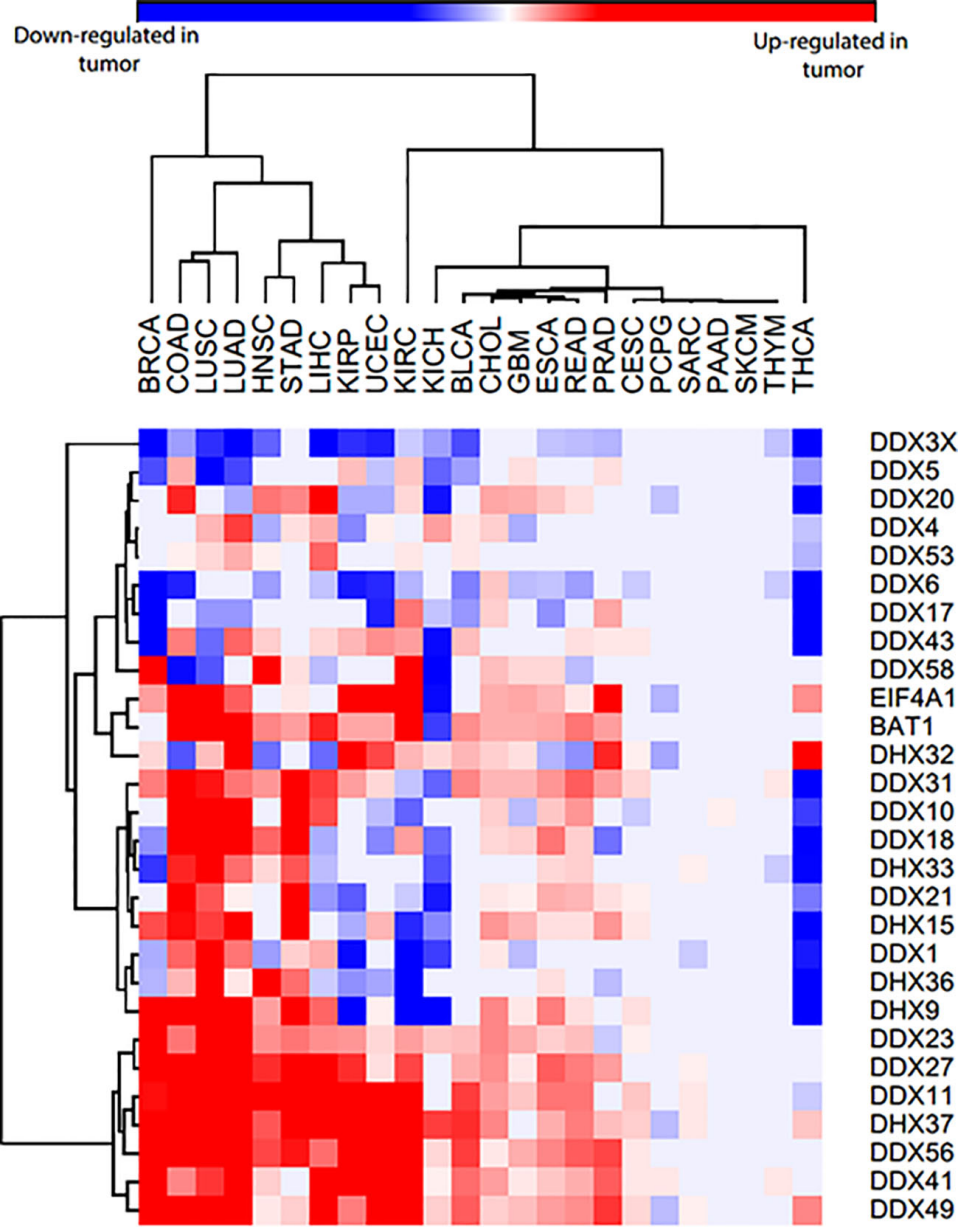
Heatmap of DEAD/H-box helicases expression in 24 different cancers and normal tissues from TCGA patient data. The blue color represents that they are significantly lost in tumor samples compared to normal samples, red color represents that they are significantly overexpressed in tumor samples compared to normal samples, and grey means no significant difference between normal and tumor samples. Cancer type abbreviations used are: BRCA, Breast invasive carcinoma; COAD, Colon adenocarcinoma; LUSC, Lung squamous cell carcinoma; LUAD, Liver hepatocellular carcinoma; HNSC, Head and neck squamous cell carcinoma; STAD, Stomach adenocarcinoma; LIHC, Liver hepatocellular carcinoma; KIRP, Kidney renal papillary cell carcinoma; UCEC, Uterine corpus endometrial carcinoma; KIRC, Kidney renal clear cell carcinoma; KICH, Kidney Chromophobe; BLCA, Bladder urothelial carcinoma; CHOL, Cholangiocarcinoma; GBM, Glioblastoma multiforme; ESCA, Esophageal carcinoma; READ, Rectum adenocarcinoma; PRAD, Prostate adenocarcinoma; CESC, Cervical squamous cell carcinoma and endocervical adenocarcinoma; PCPG, Pheochromocytoma and paraganglioma; SARC, Sarcoma; PAAD, Pancreatic adenocarcinoma; SKCM, Skin cutaneous melanoma; THYM, Thymoma; THCA, Thyroid carcinoma. BAT1 is DDX39B and EIF4A1 is DDX2.

Given that there is only sporadic data available for each of these helicases, we examined the expression of these helicases in 24 different cancer types, using gene data from patient samples available in The Cancer Genome Atlas (TCGA). This revealed that few of the helicases are always lost across the cancers examined, such as DDX3X and DDX6. Some of them are only overexpressed across human cancers, such as DDX27, DDX41 and DDX56, while most of them are both overexpressed and lost in different cancer types (**Figure 2**). We noticed few inconsistencies between the TCGA data and published findings for some helicases, which might be due to analyzing cell lines instead of patient samples (68, 134, 135). In fact, inconsistencies between data obtained from cancer cell lines and cancer patient tissues are not uncommon and have been reported (136, 137).

Apart from differential expression of these genes, mutations in DEAD/H-box helicases have also been associated with various cancers. For example, somatic mutations in DDX3X are identified in various cancers (138). Mutations in DDX18 (86) or DDX41 (139) are found in AML/MDS patients and germline mutations in RIG-I (DDX58) are found in colon cancer (110). Many missense mutations are enriched in two conserved RecA-like domains (Ref 85–88, TCGA and COSMIC data), and some frameshift mutations completely remove or drastically alter these domains, which impair the ATPase/helicase activity in these proteins. However, variants can also be found in the N- and C-terminal domains; the functional consequences of these mutations remain undetermined. Pharmacological inhibition of these helicases that are overexpressed in cancer may benefit the patients; however, identification of the targeted helicases and development of the compounds are still in their infancy (134, 140–142). Moreover, it is unclear if the inhibition of these molecules may affect normal cell functions. Application of SL or SDL may circumvent these challenges.

## Synthetic lethality and synthetic dosage lethality

**Figure 3:**
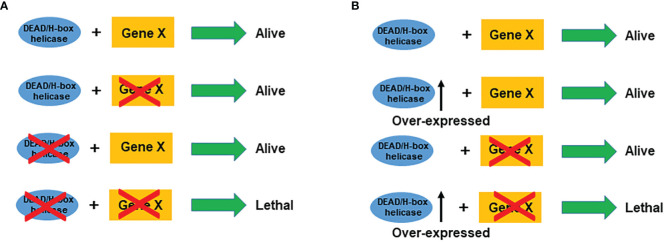
Schematic illustration of synthetic lethality **(A)** and synthetic dosage lethality **(B)** for DEAD/H-box helicases. Cross on a gene stands for mutation or inhibition.

SL represents functional relations between pairs of genes whose concomitant alteration-of-function is lethal (8). SL in DNA damage repair pathways is a promising strategy for DNA damage response. BRCA1 and BRCA2 play pivotal roles in homologous recombination (HR) repair that enable precise repair of DNA double-strand breaks (DSBs) using sister chromatids as templates (143). PARP-1 is an abundant nuclear protein in cells and plays a vital role in repairing single-strand breaks (SSBs) (144). PARP inhibitors (PARPis), which repress the catalytic activity of PARP, lead to tumor-specific cell death due to the combined deficiency in the HR and SSB repair pathway evoked by BRCA1/2 mutations and PARP inhibition respectively (145). PARPis are the first and most successful drugs designed to exploit the concept of SL (9), which provides a novel strategy for targeting other genes. In this regard, DEAD/H-box helicases, whose expression is lost in cancer, would provide a ‘tumor-specific context’ in which a second gene (SL partner) becomes a ‘vulnerable target’ that can be used to eliminate cancer cells (**Figure 3A**).

Synthetic dosage lethality (SDL), a variant of SL, decreases cell viability only when a gene is overexpressed and a second gene is inactivated (146). For DEAD/H-box overexpressed cancers, inhibition of its interaction partner will cause lethality in the cancer cells only (**Figure 3B**). Using the SDL genetic approach, a genome-wide SDL screen identified a deletion in the histone deacetylase, RPD3 gene, as selectively sensitive to the overexpression of yeast TDP1, a tyrosyl-DNA-phosphodiesterase (147). The SDL interaction was conserved in a human rhabdomyosarcoma cell line with innate TDP1 levels, and these cells were sensitive to the treatment with histone deacetylase inhibitors (147). However, so far, no SDL approach has been utilized to target DEAD/H-box helicases.

## Synthetic lethal interactions of DEAD/H-box helicases

Although SL/SDL interactions between gene pairs in cancer biology have been viewed from the target perspective, these genetic approaches also have been extensively applied in yeast to discover functional relationships, as they conserve their functions across organisms. Accordingly, we highlight several DEAD/H-box helicases below, that have been studied in model organisms as well as in human cells for synthetic lethal interactions (**Table 2**).

**Table 2:** Synthetic lethality and synthetic dosage lethality interactions of DEAD/H-box helicases.

Helicase	Targets identified	Cell line and system used	Notes	Ref.
*DDX3X*	PARP	Human BRCA1 pro- and deficient breast cancer cell lines		(148)
*DDX9* (*Dbp9*)	dbp6	*S. cerevisiae*		(149)
*DDX10* (*Dbp4*)	α-synuclein	*S. cerevisiae*, HEK293	SDL used: overexpression of dbp4 leads to synthetic lethality	(150)
*DDX11* (*ChlR1*)	Anaphase promoting complex or cyclosome (APC/C)	WBS patient fibroblast		(151)
	ESCO2	Human WBS and RBS patients’ fibroblasts and RPE1, yeast, and chicken DT40 cells	RBS: Roberts Syndrome	(152, 153)
*DDX41*(*SACY-1*)	*mog-2, emb-4, Y111B2A.25*	*C. elegans*		(154)

### DDX3

DDX3 has two paralogs, DDX3X on the X-chromosome and DDX3Y on the Y-chromosome. Sharing more than 90% identity, they are redundant for protein synthesis (155), cell proliferation and survival (156), and temperature-sensitive in hamster cell line (157), but distinct for liquid-liquid phase separation, dissolution, and translation repression (34). Interestingly, the combination of DDX3 inhibitor RK-33 and PARP inhibitor Olaparib causes SL in BRCA1-proficient breast cancer (148). DDX3X is reported to be actively recruited to sites of DNA damage in live cells (44), and it regulates the expression of DNA repair genes (158), indicating DDX3X is essential for DNA repair, such as non-homologous DNA end joining (NHEJ). RK-33 is a small molecule that binds DDX3X and inhibits its helicase activity (134). Mechanistically, RK-33 might inhibit NHEJ repair. Combination of RK-33 and PARPis, which inhibit DDX3X and PARP and block SSB and NHEJ repair pathways respectively, leads to cell death, representing a classical ‘between-pathway’ SL interaction. Whether RK-33 can manipulate any other SL interactions of DDX3X for better outcomes requires further investigation. More recently, analyses of multiple-omics datasets and experimental validation revealed that redundancy exists between DDX3X and DDX3Y (159); therefore, we have to consider the gender of the patient and look for any loss of chromosome Y in male patients before applying RK-33. In addition, it remains to be determined, whether RK-33 binds and inhibits the DDX3Y protein in addition to DDX3X.

It is worth noting that DDX3 has been reported to function as both an oncogene and a tumor suppressor. DDX3 exerts oncogenic roles in glioblastoma (160, 161), meningioma (162), Ewing sarcoma (68), prostate cancer (134), chronic lymphocytic leukemia (163, 164), pancreatic ductal adenocarcinoma (165), and gallbladder cancer (126). In contrast, DDX3 acts as a tumor suppressor in natural killer/T-cell lymphoma (166) and cutaneous squamous cell carcinoma (20). Moreover, the dual role of DDX3 have been reported in same type of cancer, including breast cancer (124, 167–169), hepatocellular carcinoma (170, 171), lung cancer (142, 172), colorectal cancer (70, 173, 174), and head and neck squamous cell carcinoma (69, 175, 176). So far, there are no mechanistic explanations for the complex behavior of DDX3 in these cancers, which appears to be context dependent. For its tumor suppressive function, particularly in cases of LOF mutations, we believe that the SL approach can be used for drug development; for its oncogenic roles, the SDL strategy could be potentially applied.

### DDX9 (Dbp9)

As mentioned above, SL interactions in yeast reveal functional relationship between gene pairs. *DDX9* (*Dbp9*), encoding an essential nucleolar protein involved in 60S-ribosomal-subunit biogenesis, exhibits an SL relation with DDX6 (Dbp6), a component required for 60S-ribosomal-subunit assembly in yeast (177). Interestingly, *dbp6/dbp9* double mutants show synthetic lethality: no viable *dbp9/dbp6* double mutants could be recovered, indicating a functional interaction of *Dbp9* with *Dbp6*, and accumulated defects in ribosome biogenesis lead to cell death (149). Our analyses of the TCGA data (**Figure 2**) show that DDX6 is downregulated in multiple cancers. If the yeast interactions are conserved, inhibition of DDX9 in these cancers may result in a therapeutically relevant SL interaction.

### DDX10 (Dbp4)

Parkinson’s disease (PD) is characterized by loss of dopaminergic neurons in midbrain and the presence of Lewy inclusion bodies, which are predominantly composed of misfolding and aggregation of the α-synuclein protein. Interestingly, a reciprocal susceptibility was observed in the PD patients towards the occurrence of melanoma (178). Although fundamentally divergent, the common link between the two disorders is the accumulation of α-synuclein into amyloid fibrils. Gerhard Braus’s group expressed human α-synuclein in yeast cells and monitored dosage-dependent toxicity effects on the formation and reduction of inclusions bodies (150). They identified the nucleolar DDX10 (yeast ortholog Dbp4) as a strong enhancer of α-synuclein toxicity. While downregulation of Dbp4 rescued cells from α-synuclein toxicity, overexpression of Dbp4 led to an SL phenotype. These findings provide a novel link between nucleolar processes and α-synuclein-mediated toxicity, with DDX10 emerging as a promising drug target for melanoma.

### DDX11 (ChlR1)

RNAi-dependent knockdown of DDX11 causes premature sister chromatid separation and a profound delay in mitotic progression of human cells, suggesting that DDX11 is required to establish proper sister chromatid cohesion during the S phase (179). Biallelic DDX11 mutations in humans cause WBS, which is characterized by severe microcephaly, pre- and post-natal growth retardation, and abnormal skin pigmentation (54). Rob Wolthuisb and his team found that DDX11 mutations cause cohesion defects in patient fibroblasts. This discovery prompted them to subject patient’s fibroblasts to a genome-wide siRNA screen to search for genes that are synthetically lethal with mutant DDX11 (151). Screening results revealed several components of the anaphase promoting complex or cyclosome (APC/C) as top hits, and the DDX11 mutant cells proved to be hypersensitive to the inhibition of the APC/C complex. Mechanistically, they found that APC/C inhibition aggravates cohesion defects and causes mitotic death. As the TCGA data indicates that DDX11 is overexpressed in multiple cancers (**Figure 2**), further analyses of this gene and the effect of its overexpression in sister chromatid cohesion might reveal if APC/C inhibition could still remain a viable therapeutic option. The same group also found that ESCO2 (establishment of cohesion 1 homolog 2) was one of the strongest hits in their siRNA screen, in which they used WBS patient fibroblasts (152). The synthetic lethality between DDX11 and ESCO2 was also observed in yeast orthologues Chl1 (DDX11/ChlR1) and Eco1 (ESCO2) (180), and chicken DDX11 and ESCO2 (153). Thus, some of the conserved interactions observed in yeast may be of high relevance in humans as well.

### DDX41 (SACY-1)

DDX41 is conserved across species; its orthologs abstrakt in *Drosophila* (181), Sacy-1 in *C. elegans* (154), and *DrDDX41* in zebrafish (182) have been studied. Both germ line and acquired somatic mutations of DDX41 have been associated with MNs, MDS and AML (62, 139). David Greenstein’s group used a sacy-1 reduction-of-function genetic background in *C. elegans*, conducted a genome-wide RNAi screen, and identified five clones that produced increased levels of sterility, gamete degeneration, or embryonic lethality (183). The five RNAi clones target the transcripts of three genes: *mog-2* (one clone), *Y111B2A.25* (one clone), and *emb-4* (three clones) (154). Because three phenotypes have been observed in a variety of sacy-1 mutant alleles, individual siRNA in sacy-1 mutant animal confirmed that genes *mog-2*, *Y111B2A.25*, and *emb-4* are synthetic lethal interactors of *sacy-1* (*DDX41*). Interestingly, similar to *DDX41*, these three genes encoded proteins that are constitutive components of the spliceosome (154), and it remains to be seen if these interactions are still conserved in humans. As DDX41 is overexpressed in multiple cancers, spliceosome inhibitors should benefit these patients, provided the functional interaction is conserved.

## Exploiting DEAD/H-box helicases for synthetic lethality approaches

Olaparib, rucaparib, niraparib, talazoparib, and veliparib, classified as PARPis, are, so far, the only SL drugs for cancer patients with BRCA1/2 mutations (9). There is an urgent need for new SL targets in other cancers. Many DEAD/H-box helicases are upregulated in various malignancies, while some of their mutations are also associated with cancers. Therefore, there is a growing interest in DEAD/H-box helicases as plausible targets for anti-cancer therapy. Indeed, identifying and developing SL interactions of DEAD/H-box helicases as therapeutic targets or a direct targeting of these helicases have been attempted (140, 150, 184, 185).

DEAD/H-box helicases have potentials to be targeted for future drug developments due to their unveiled ties with cell toxicity in various cancers (52). The lethal characteristic of helicases becomes more prominent upon their inhibition in overexpressing cancers. Upregulation of DDX5 leads to poor patient outcomes through the promotion of tumorigenesis and tumor recurrence (74). Consequently, depletion of DDX5 leads to the suppression of the mammalian target of rapamycin complex 1 (mTORC1) signaling pathway and induces apoptosis in prostate cancer cells (184). Compounds targeting DDX5, such as Resveratrol and RX-5902 (186), have been developed; however, further studies are required to evaluate anti-cancer effectiveness of these drugs. DHX9 is overexpressed in lung cancer (187) and its suppression is selectively lethal to cancer cells, but is tolerable for normal cells (188). Active screening for DHX9 inhibitors is in progress, as they can potentially be used as therapeutic agents specifically attacking cancer cells (140).

Another exciting prospect is to identify SL and SDL partners that have already been implicated in cell apoptosis in a cancer-specific manner or whose DEAD/H-box helicase partners, when overexpressed, have a putative apoptotic nature. An elevated expression of DDX10 in cancer leads to a poor survival rate in chondrosarcoma patients (78). Additionally, inhibition of α-synuclein displays tumor growth suppression in melanoma cells (189). Since DDX10 is a strong enhancer of α-synuclein-induced toxicity (150), we can employ SDL interaction between DDX10 and α-synuclein and use α-synuclein inhibitors as promising therapeutics for cancer, such as melanoma. Fortunately, various drugs used in PD’s model follow the same principle of α-synuclein inhibition and can be evaluated for cancer therapy (185). However, this may be context dependent as DDX10 is found to be both overexpressed as well as lost in cancers (**Figure 2**).

Many DEAD/H-box helicases, such as DDX3, DDX5, DDX10, and DDX21, have been reported to act both as oncogenes and tumor suppressors in different contexts. Therefore, in-depth research is essential to understand the intricacy and interplay of their dual antagonistic roles, and to ultimately, to effectively use them as targets for cancer treatment. Generally, the SDL approach might be useful in cancers with over-expressed DEAD/H-box helicases that function as oncogenes, while the SL approach might work with DEAD/H-box helicases that act as tumor suppressors. In both scenarios, the outcome is to promote cancer-specific apoptosis through pharmacological interventions. Identifying and manipulating these interacting partners open new avenues for drug development in cancer-targeted therapy.

Paralog genes arise from gene duplication, an evolutionary mechanism for creating new genes, which result in two functionally distinct genes, or more frequently, functionally overlapping genes. In fact, 13700 or two-thirds of human protein coding genes are paralogous (190). Paralogs provide both unique opportunities and challenges for the SL approach in developing targeted therapies. If a single gene is depleted, its paralog can compensate by taking over its function (191). Thus, the loss of a single gene is well tolerated by the cell; this phenomenon is called paralog buffering. However, if both paralogs are depleted, there is no mechanism in place to compensate for the lost function and this results in cell lethality (190). Recent CRISPR-based screenings and mining of publicly available data have identified several SL interactions among paralogs (159, 190–194). Paralog dependency is found in CSTF2-CSTF2T, DNAJC15-DNAJC19, FAM50A-FAM50B, and RPP25-RPP25L (159), CCNL1-CCNL2, CDK4-CDK6, MEK1-MEK2, and OXSR1-STK39 (190), CNOT7-CNOT8, COPS7A-COPS7B, CCNE1-CCNE2, and CCNT1-CCNT2 (192), STK38–STK38L and TET1–TET2 gene combinations (194). Paralog redundancy has been identified in CCNL1-CCNL2, OXSR1-STK39, EIF1-EIF1B, G3BP1-G3BP2, GFPT1-GFPT2, and PDS5A-PDS5B (190), MAP2K1-MAP2K2, RAS-RAF, FAM50A-FAM50B (192), sex chromosome genes ZFX-ZFY, DDX3X-DDX3Y, EIF1AX-EIF1AY (159), SAR1A–SAR1B, RAB1A–RAB1B, LDHA–LDHB, RBM26–RBM27 and hnRNPF–hnRNPH3 gene pairs (194) Recently, it was reported that VRK1 is a SL target in VRK2-mutated or silenced cancers (195, 196) and SMARCA2 is a SL target in SMARCA4 mutated cancers (197), suggesting SL is an excellent approach for paralog-related cancer treatment. Although ARID1A and ARID1B are synthetic lethal (198), it was reported that dual ARID1A-ARID1B loss leads to rapid carcinogenesis (199). This finding emphasizes that caution should be executed when developing new paralog-directed SL therapies. Lastly, some genes have more than two paralogs, such as Akt1-Akt2-Akt3 and RAD51-RAD51B-RAD51C-RAD51D-XRCC2-XRCC3, while FRG1 has 23 paralogs (200), which should present challenges to implementing the SL approach.

Multiple paralogues exist in the DEAD/H-box helicase family, including DDX2A-DDX2B, DDX3X-DDX3Y, DDX19A-DDX19B, DDX60-DDX60L and DDX39A-DDX39B. The DDX19A-DDX19B paralog pair engages in the SL interaction, where enhanced DDX19A expression is strictly required as a compensatory response to the low level of DDX19B (191, 193). Besides the DDX3X-DDX3Y and DDX19A-DDX19B pairs, the remaining paralog pairs have not been studied. Thus, computational predictions combined with experimental validation are expected to expand the horizon of uncovering paralog genetic interactions among helicase genes that can be used as potential therapeutic targets.

## Conclusions and future perspectives

Although various small molecules have been utilized to target patient-specific molecular alterations for personalized cancer treatment protocols, their efficiency rate is still far from the desired. A drawback we currently face with the personalized treatments is their off-target effects, as these targeted therapeutics fail to effectively differentiate normal cells from the cancerous ones, ultimately leading to cytotoxicity. As we solve the structure-function relationship and interaction network of various DEAD/H-box helicases, it appears that we cannot limit their function as only molecular motors for nucleotides. LOF and overexpression of DEAD/H-box helicases in specific cancers are likely to provide an effective platform for developing more selective treatment approaches by inhibiting SL/SDL partners of these molecules, thus revolutionizing the arena of personalized genotype-based targeted therapeutics. Combination therapy of SL/SDL drugs with known chemotherapeutics might also synergistically improve patient outcomes. These approaches may provide a better window for therapeutic index optimization and minimize undesirable off-target effects associated with drug administration. It can also further optimize patient-specific treatment plans by targeting genetic vulnerabilities associated with their specific mutations in certain cancer subtypes and design solutions to LOF mutations that obviously cannot be targeted by our traditional approaches.

Despite the recent limelight and efforts in SL target discovery, there are only a handful of success stories in SL drug development reaching clinical trials. A molecule targeted by an SL drug may deregulate multiple biological processes, as different pathways share components, leading to more adverse patient outcomes. Thus, more in-depth understanding of the mechanisms is essential to unveil the complexities and heterogeneity of the SL interactome to pinpoint the molecular network and dependencies across various cancer types. The discovery of SL/SDL interacting compounds to enhance drug selectivity and design new effective combination therapies will make this process even more exciting. By investigating DEAD/H-box helicases within the SL/SDL context and implementing the generated knowledge, we can accelerate the process of bringing novel drugs to the bedside and positively impact cancer patient outcomes.

## Author contributions

AA: Conceptualization, Data Curation, Writing, Visualization. HP and RS: Data Curation, Revision. FSV: Data Curation (TCGA). AK and AF: Revision. FJV and YW: Conceptualization, Data Curation, Writing and Editing, Visualization, Supervision, Funding acquisition. All authors contributed to the article and approved the submitted version.
